# Thioflavin T—a Reporter of Microviscosity in Protein Aggregation Process: The Study Case of α-Synuclein

**DOI:** 10.1021/acs.jpclett.4c00699

**Published:** 2024-06-20

**Authors:** Konstantin Rusakov, Aadil El-Turabi, Lasse Reimer, Poul Henning Jensen, Piotr Hanczyc

**Affiliations:** † Faculty of Construction and Environmental Engineering, Warsaw University of Life Sciences, 02-776 Warsaw, Poland; ‡ University of Oxford, Jenner Institute, Nuffield Department of Medicine, OX3 7DQ Oxford, U.K.; § DANDRITE, Department of Biomedicine, Aarhus University, 8000 Aarhus, Denmark; ∥ Institute of Experimental Physics, Faculty of Physics, University of Warsaw, Pasteura 5, 02-093 Warsaw, Poland

## Abstract

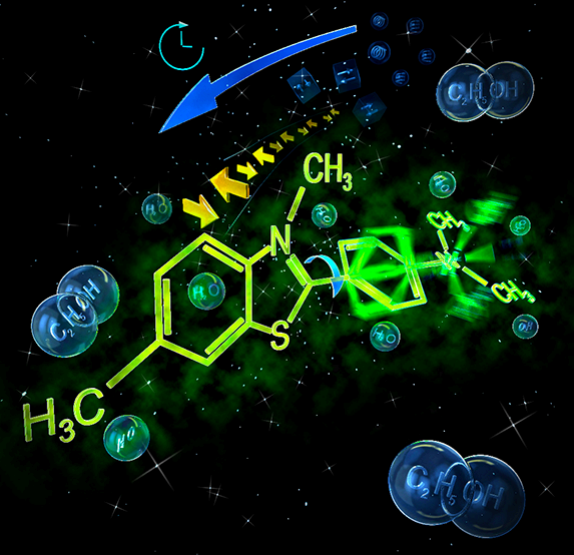

Thioflavin T (ThT) informed microviscosity changes can be used to monitor protein aggregation. Steady-state, time-resolved and lasing spectroscopy were used to detect transient states in α-synuclein - a protein associated with Parkinson’s disease. The major focus was on the nucleation phase, where conventional ThT fluorescence assay lacks appropriate sensitivity to detect early stage oligomers. Instead, lasing spectroscopy and lasing threshold parameters, in particular, were sensitive to detecting protein oligomers. Through lasing spectroscopy, a change in microviscosity correlating with the stages of protein aggregation was observed at two wavelengths 405 and 440 nm. The two wavelengths are associated with free dye molecules and β-sheet bound ThT molecules. This provides a perspective on elucidating the early formed protein aggregation, a critical aspect in understanding the pathogenesis of neurodegenerative diseases. The insights from the presented study shows the potential of using lasing spectroscopy as a sensitive tool in studying protein aggregation dynamics.

Protein aggregation is a pivotal pathogenic process in neurodegenerative diseases.^1−4^ However, the mechanisms by which the aggregation contributes to neurodegeneration are not fully elucidated.^5−8^ In this study, dye doped α-synuclein, lysozyme, and insulin were utilized to investigate microviscosity in protein aggregation process, probed by lasing effect.^9^ α-Synuclein served as the primary model system for detailed analysis, which, in addition to lasing, was characterized using steady-state and time-resolved fluorescence techniques.

For probing the transition of proteins during aggregation, Thioflavin T (ThT) staining is regarded as the gold standard.^10−12^ ThT exhibits a marked increase in fluorescence when its molecules are confined within the grooves formed by β-sheets in the fibrillary structure of amyloids.^13^ In contrast, the absence of longitudinal grooves in native protein configurations or in oligomeric states (lacking β-sheets) results in negligible ThT fluorescence, underscoring the dye’s insensitivity to these protein forms.^14^ This differential fluorescence response facilitates the discernment of amyloid fibrils from nonaggregated proteins.^15^

Besides using ThT in protein aggregation studies, this dye is also recognized as a viscosity reporter, characterized by a low fluorescence in environments of low viscosity and heightened fluorescence in more viscous media.^16^ This behavior is akin to protein binding, attributed to the linkage with hindering intramolecular rotation of ThT rings.^17,18^ Thus, in the realm of protein aggregation research, a clear distinction between the fluorescence effects of ThT arising from its binding to protein aggregates and changes in viscosity remains ambiguous. While not demonstrating strong fluorescence enhancement, the latter effects could indicate structural transformations during protein aggregation. These transformations will likely alter microviscosity, as larger oligomers present a more restricted environment for ThT, and some nonspecific intermolecular interactions can occur between positively charged ThT and negatively charged amino acids in proteins.^19^

Herein, we propose applying lasing spectroscopy in Fabry–Perot cavities to probe protein structures in the condensed phase to enhance emission arising from viscosity. The Fabry–Perot cavity lasing technique utilizes a pair of mirrors functioning as photonic resonators, which amplify light within the cavity.^20,21^ The protein-ThT solution sandwiched between the mirrors is the gain medium. Figure 1a represents the illustration of the experimental setup for measuring lasing and enlarged image showing the pair of mirrors with protein-ThT solution inside.

**Figure 1:**
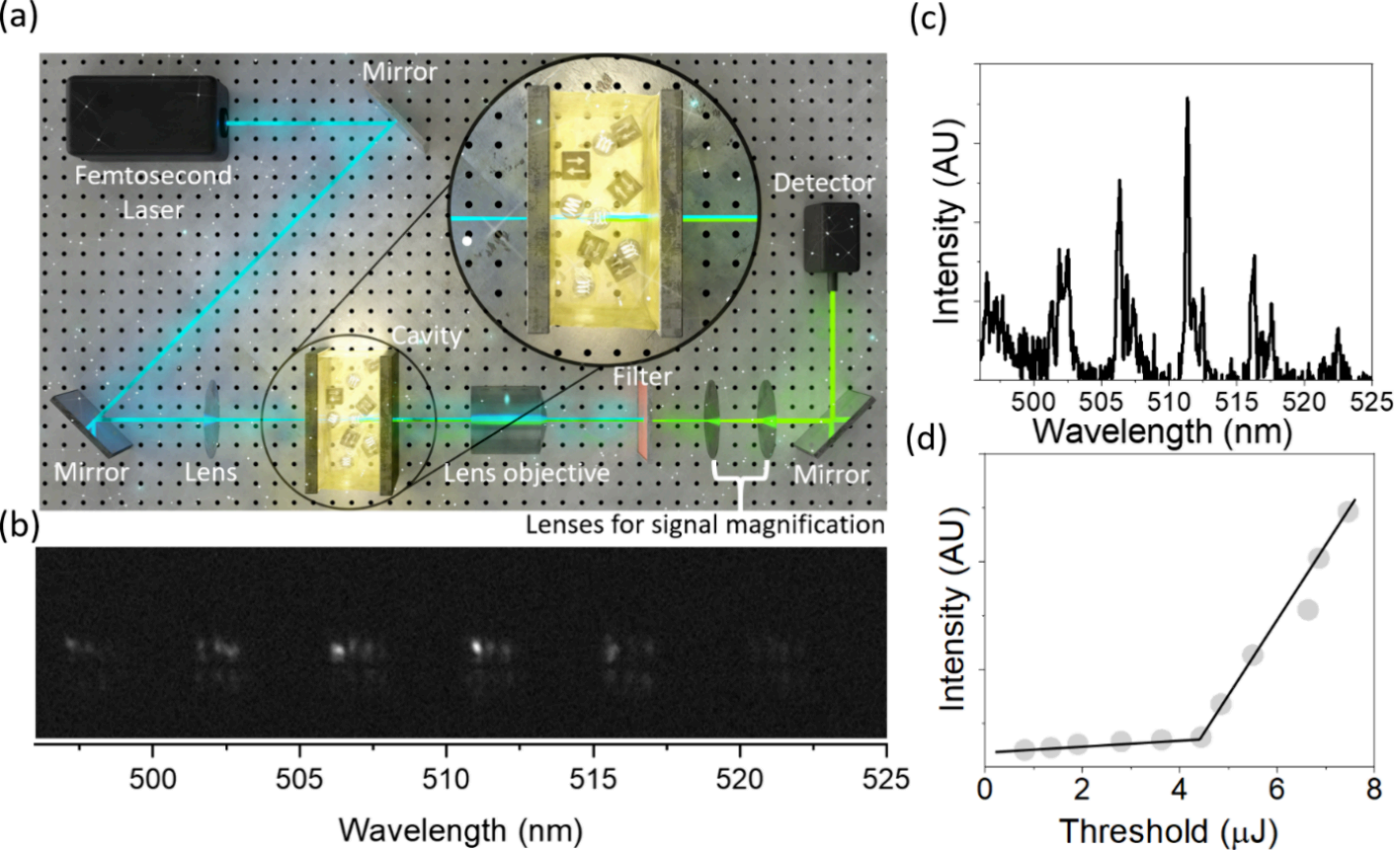
Thioflavin T lasing characterization. (a) Schematic representation of the experimental lasing setup (inset, an enlarged image of mirror cavity) with α-synuclein stained with ThT. (b) Lasing measurement output, evident as bright spots, indicates lasing activity surpassing threshold levels (λ_ex_ = 405 nm). (c) The resolved lasing spectrum from (b) displays multiple lasing peaks in Thioflavin T dissolved in ethanol. (d) The relationship between pump energy and emitted intensity highlights the exponential increase in intensity upon reaching the lasing threshold.

The amplification process is initiated when the energy within the cavity exceeds the threshold necessary for population inversion, culminating in the emergence of bright spots in the camera and distinct lasing peaks in the reformatted spectrum Figure 1(b, c). The specific pump energy at which this transition manifests is referred to as the lasing threshold^22^ (Figure 1(d)).

Two distinct excitation wavelengths -405 and 440 nm - were employed to probe free and bound dye molecules, respectively.^23,24^ The rationale behind utilizing the 405 nm excitation wavelength was to leverage free dye as a reporter for viscosity and to assess weak, nonspecific intermolecular interactions between ThT and proteins: insulin, lysozyme and α-synuclein in various states (Figure S1).

A distinct discrimination between macroviscosity and microviscosity remains ambiguous.^25,26^ Macroviscosity is easier to understand because it can be described as measuring a fluid’s resistance to flow at the macroscopic scale.^27^ ThT, which is a molecular rotor, can be elegantly used to study viscosity because the dye’s rotational dynamics respond to the fluid resistance. Thus, at first, studies of macroviscosity’s influence on ThT lasing characteristics were performed across a gradient of solvent viscosities, encompassing water, ethanol, butanol, and ethylene glycol. The influence of macroviscosity on ThT lasing in different solvents was used to understand the microviscosity effects on lasing in ThT mixed with proteins with the focus on model protein: α-synuclein. In the lasing experiments, the gain medium (ThT dissolved in various solvents), is enclosed between two mirrors that create a lasing cavity. Using a cavity system, lasing thresholds of ThT were determined in three solvents - ethanol, butanol, and ethylene glycol. Notably, no lasing phenomena were observed in ThT solutions prepared in water or phosphate-buffered saline (PBS) (Table 1).

**Table 1:** Determination of the Lasing Thresholds in Thioflavin T Dye at Two Wavelengths 405 and 440 nm

	Viscosity 10^–3^ Pa s^a^	Dye concentration for lasing (mM)	Lasing thresholds (μJ) excited at 405 nm	Lasing thresholds (μJ) excited at 440 nm
Water	0.89	78.4^b^	-	-
Ethanol 96%	1.08	25	-	-
Ethanol 99%	1.19	25	4.52	-
Butanol	2.59	25	3.91	-
Ethylene glycol	16.1	18.8	1.98	3.24

a Solvent viscosity values: http://murov.info/orgsolvents.htm. (date of access: 15.03.2024).

b Saturated concentration of ThT in water.

Lasing thresholds were evaluated under two excitation wavelengths: 405 and 440 nm. These two wavelengths were chosen to correlate with protein aggregation processes that will be described in the next sections. Excitation with 405 nm is typically associated with unbound ThT, and 440 nm correlates with ThT bound to amyloid fibrils.

It was observed that at an excitation wavelength of 405 nm, the lasing thresholds of ThT exhibited a decreasing trend in concurrence with the rising viscosity of the solvent. When the dye molecules were excited at 440 nm, the lasing threshold was discernible solely in the ethylene glycol solvent. It confirms that free ThT molecules exhibit lasing predominantly at 405 nm when in a low-viscosity solvent, making lasing an excellent tool for studying microviscosity in ThT-stained protein aggregates.

Building on this conceptual framework, our study adopts the term ’microviscosity’ to delve into the intricate, nonspecific interactions between proteins and ThT in the early stage of aggregation when β-sheets structures are yet to form. This terminology facilitates a more nuanced understanding of the dynamic interplay between protein units and dye interaction emphasizing the important role of microenvironment around ThT.

Prior to investigating the lasing effects in ThT mixed with α-synuclein, fluorescence kinetics and time-resolved studies were conducted, focusing particularly on the nucleation phase of α-synuclein (Figure 2). First, α-synuclein was dissolved in PBS and ultracentrifuged using filter columns in order to remove any preaggregates. The nucleation phase was induced in the filtered fraction by agitation at 900 rpm for 10 min and then sample was maintained at a constant temperature of 37 °C. After that treatment the nucleation phase was lasting approximately 10 h. Emission spectra were obtained upon excitation at 405 and 440 nm, revealing relatively weak fluorescence intensities at both wavelengths. This observation is characteristic of the nucleation phase in protein aggregation, where unbound dye molecules predominantly deactivate through the Twisted Intramolecular Charge Transfer (TICT) mechanism. A comparative analysis of pure ThT in water and ThT mixed with α-synuclein after 30 min indicated a marginally increased fluorescence in the latter (inset of Figure 2(a)). This disparity in emission intensities between ThT in water and ThT-α-synuclein was consistent throughout the nucleation phase (Figure 2(b, c)). It is hypothesized that, in the early stages of aggregation, where β-sheet structures have not yet formed, this difference may be attributable to alterations in the local microviscosity surrounding the dye molecules. To further investigate the concept of microviscosity, fluorescence lifetimes of ThT were measured at the onset of the nucleation phase (Figure 2(d, e)). These measurements confirm the detectability of ThT signals from the early stages of protein aggregation. However, approximately 40% of the fluorescence decay occurred below the resolution of our instrumentation (below the instrument response function (IRF)), suggesting a significant rotational freedom of the dye’s molecular rings. This rotation leads to ultrafast nonradiative relaxation, which is eventually restricted upon the formation of fibrils during the elongation phase. Significantly prolonged fluorescence lifetimes were observed at elevated temperatures after 120 h of incubation.

**Figure 2:**
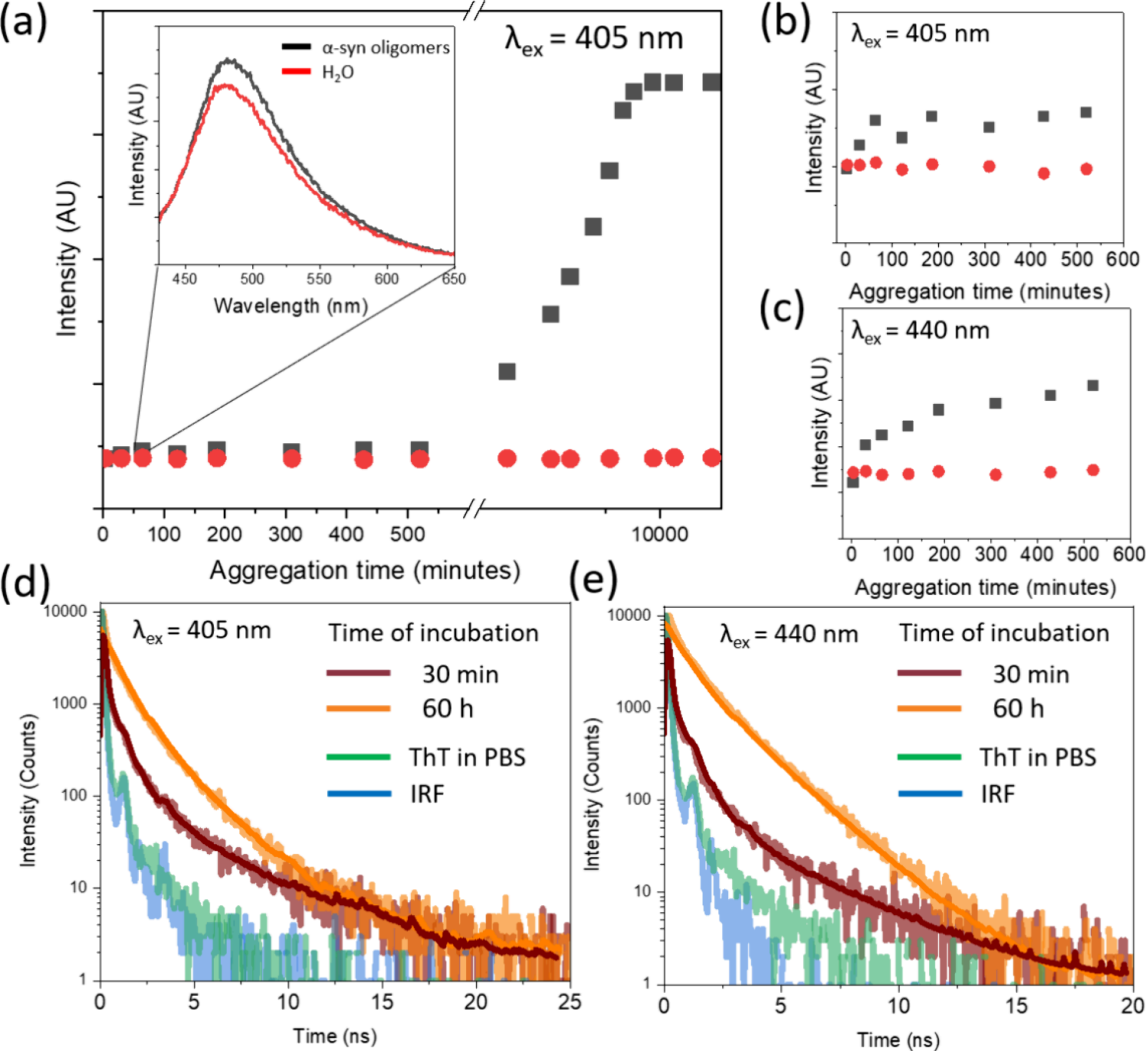
Steady-state and time-resolved fluorescence of ThT-Stained α-synuclein (a) fluorescence kinetics of Thioflavin T (ThT) in the presence of α-synuclein (black squares). The initial measurement was performed on an ultracentrifuged sample to eliminate preaggregates. Subsequently, the sample was agitated for 10 min and placed in a fluorescence spectrometer’s chamber at 37 °C. For comparison, fluorescence kinetics of ThT in PBS was also measured under identical conditions. (b, c) Zoomed-in view of the fluorescence kinetics, focusing on the nucleation phase. Black squares represent ThT with α-synuclein, while red dots indicate the fluorescence peak of pure ThT in water. Measurements were conducted with excitations at 405 nm (b) and 440 nm (c). (d, e) Fluorescence decays of ThT with α-synuclein at two different stages: after 30 min (brown), representing early aggregation and after 60 h (orange), when mature fibrils had formed. For comparison, decay curves of pure ThT (green) and the instrument response function (IRF, blue) are presented for excitations at 405 nm (d) and 440 nm (e).

In the initial stages of the nucleation phase, it is noteworthy that the average fluorescence lifetimes of ThT are remarkably similar when excited at 405 and 440 nm, being 0.84 and 0.82 ns respectively (table. 2). This observation indicates an absence of specific interactions between the dye molecules and the amorphous α-synuclein preaggregates during this phase, implying that microviscosity is the predominant factor influencing the rotational dynamics of ThT. In contrast, mature fibrils exhibit a biexponential decay profile. Notably, at 405 nm, approximately 70% of the decay is attributed to a short-lived component with a lifetime of 0.82 ns, which aligns closely with the average lifetime recorded at the nucleation phase’s onset, underscoring the impact of microviscosity. At 440 nm, the longer-lived component dominates, accounting for over 50% of the decay, suggesting an effective steric hindrance imposed on ThT within the β-sheet grooves of the fibrils.

**Table 2:** Fluorescence Decay Times (τ_i_) of ThT-Stained α-Synuclein Obtained by Fitting Two- and Three-Exponential Functions to Fluorescence Decays^a^

	405 nm				440 nm			
	τ_1_ (ns)	τ_2_ (ns)	τ_3_ (ns)	τ_avg_ (ns)	τ_1_ (ns)	τ_2_ (ns)	τ_3_ (ns)	τ_avg_ (ns)
α-synuclein	0.06^b^	0.63	2.84	0.84	0.04^b^	0.65	3.42	0.82
**spin filtered**	±0.01	±0.01	±0.01		±0.01	±0.01	±0.01	
Time 30 min	(0.36)	(0.45)	(0.19)		(0.39)	(0.47)	(0.14)	
α-synuclein	-	0.82	2.05	1.18	-	0.70	1.90	1.33
**spin filtered**		±0.01	±0.01			±0.01	±0.01	
Time 120 h		(0.71)	(0.29)			(0.48)	(0.52)	

a The deconvolution of the experimentally measured instrument response function (IRF) was accounted for measured decay (for details, see the Supporting Information). The parameter τ_avg_ represents the amplitude-weighted average fluorescence lifetime.

b Signal below the IRF.

To further explore the hypothesis that microviscosity influences the fluorescence characteristics of ThT, lasing experiments were conducted in a mixture of water and ethanol. As depicted in Figure 1, ThT dissolved in 99% ethanol exhibited lasing activity (ethanol viscosity: 1,19 × 10^–3^ Pa *x* s),^28^ whereas no signal was detected when the ethanol was diluted to 96% using water. This difference between the two samples, which vary by addition of small amount of water, highlights strong sensitivity of ThT to its microenvironmental conditions. The absence of lasing in 96% ethanol suggests that water molecules preferentially form a protective shell around the dye molecules hindering the access to ThT by ethanol solvent. This arrangement promotes ultrafast nonradiative decay channels, thereby inhibiting the population inversion necessary for laser action. It indicates that ThT is a sensitive molecular probe of subtle changes in the microenvironment.

In pursuit of understanding the influence of microviscosity on ThT fluorescence enhancement, lasing spectra and thresholds were assessed during the initial stages of lysozyme, insulin and α-synuclein aggregation. For the lasing experiments the stock solution of lysozyme was 360 mg/mL. Insulin and α-synuclein were concentrated using column filters of 3 kDa pore size by 10-fold reduction of the initial solution volume. The protein concentration was estimated to be 100 mg/mL for insulin and 120 mg/mL for α-synuclein. Increased concentration is intensifying the microviscosity effects due to the compaction of protein-ThT material and lasing effects were observed in each protein aggregated for 30 min.

Figure 3 presents lasing thresholds for ThT in combination with α-synuclein, maintained at 37 °C over the course of the experiment in a sandwiched mirror cavity setup. Lasing measurements were conducted at intervals corresponding to the aggregation timeline of α-synuclein, aligning with the fluorescence kinetics depicted in Figure 2(a). Initial measurements identified lasing thresholds at 0.55 μJ for 405 nm and 1.1 μJ for 440 nm excitation wavelengths. Remarkably, within the first 100 min, the thresholds remained stable at 405 nm, while a significant reduction was observed at 440 nm, reaching a minimum energy required for population inversion at the level of 0.46 μJ.

**Figure 3:**
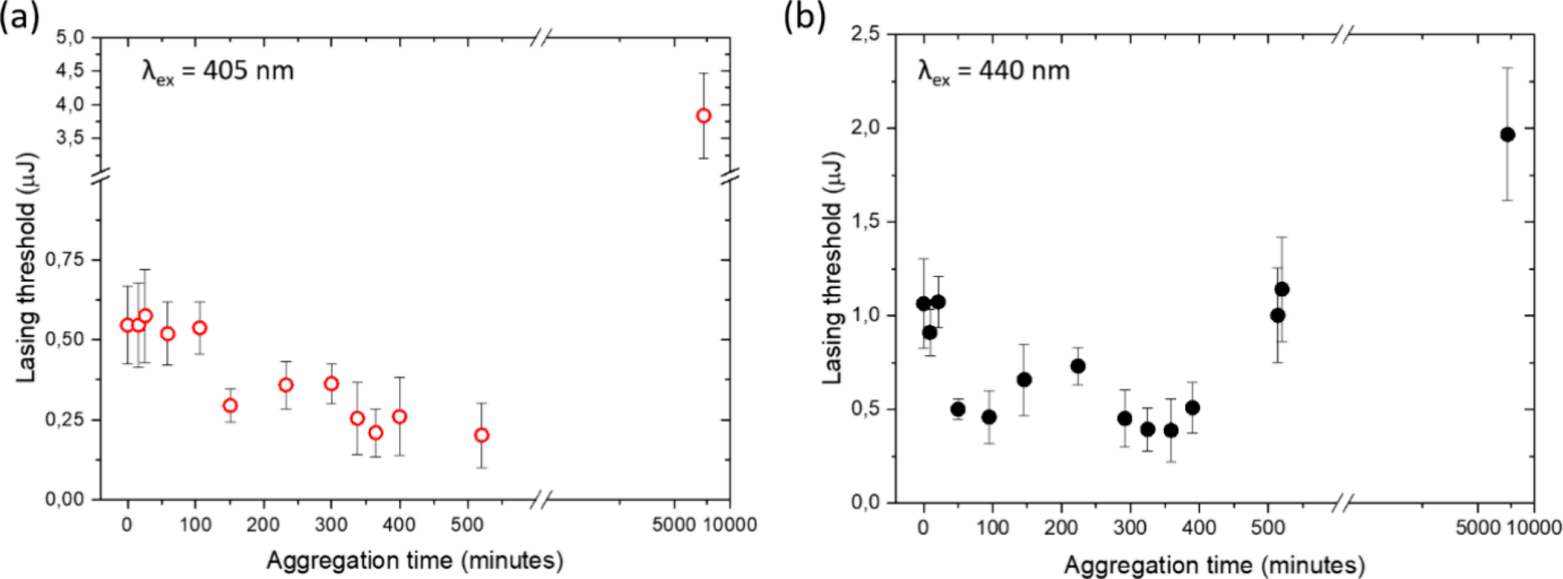
Kinetic analysis of lasing thresholds in ThT-stained α-synuclein. (a) Lasing threshold kinetics for α-synuclein samples stained with Thioflavin T (ThT) and excited at 405 nm, represented by red open circles. (b) Corresponding kinetics for samples excited at 440 nm, depicted with black filled circles. The concentration of α-synuclein was maintained at 8.3 mM, while ThT concentration was 26.1 mM. Measurements were taken at predefined time intervals. The experiment was conducted in triplicate, and the dots in Figure 3 display the average lasing thresholds, with error bars indicating the variability across the three repetitions.

Subsequent observations between 100 and 500 min indicated a gradual decreasing trend in lasing thresholds at 405 nm, while fluctuating thresholds were determined when exciting at 440 nm. More specifically, upon excitation ThT at 440 nm, the thresholds initially increased between 120 and 250 min, followed by a notable reduction to 0.39 μJ after 300 min at 37 °C. Subsequently, a significant increase in lasing thresholds was observed from 500 to 550 min, reaching approximately 1 μJ. This rise coincides with the completion of the nucleation phase around 500 min, as illustrated in fluorescence kinetics Figure 2(a), suggesting the initiation of protofibril formation. Notably, during the 500–550 min interval, the lasing threshold studies at 405 and 440 nm yielded divergent results; the increase in viscosity likely predominated the dye behavior at 405 nm, whereas the ThT molecules excited at 440 nm were more affected by binding to protofibrils and enhanced light scattering on a microscale.

The lasing threshold data upon 405 nm excitation suggest a gradual increase in microviscosity correlated with the aggregation timeline. Concurrently, the variable thresholds at 440 nm indicate that ThT undergoes rearrangements in response to the transient states of the aggregates, a phenomenon typical in the early nucleation stage of proteins, where aggregates can form and reform.^29^ The lasing experiments at 440 nm revealed that ThT conformation is sensitive to these transient aggregate structures. Although it is widely acknowledged that ThT exhibits weak emission in the nucleation phase due to the absence of binding to protein monomers and oligomers, the lasing behavior in optical cavities provides compelling evidence that protein microviscosity environment significantly influences dye conformation and facilitates weak, nonspecific intermolecular interactions between ThT and the protein amorphous aggregates.

The experiments detailed above show a significant advantage of lasing analysis over traditional fluorescence techniques. Specifically, the lasing thresholds observed during the nucleation phase of α-synuclein demonstrate a time-dependent decrease. This trend not only mirrors the progressive state of aggregation but also indicates an increase in microviscosity. In contrast, traditional ThT fluorescence assay remains nearly undetectable during the nucleation stage, highlighting the utility of lasing thresholds as a tool to monitor the entire aggregation process, from the initial stages to the formation of mature fibrils. As the aggregation process culminates in fibril formation, the binding dynamics of ThT shift from nonspecific interactions due to microviscosity to a preference for binding within the β-sheet grooves of the fibrils. This transition was evidenced in lasing threshold experiments, where, on the fifth day of incubation at 37 °C, the thresholds were recorded at 3.84 μJ and 2 μJ for excitation wavelengths of 405 and 440 nm, respectively. These findings suggest that ThT molecules bound to fibrils provide more effective optical feedback for lasing compared to freely excited molecules at 405 nm. Moreover, the general increase in lasing thresholds at both excitation wavelengths is indicative of the strong light-scattering properties of fibrils, which adversely affect lasing.^30^ Collectively, these results reveal that the aggregation process impacts lasing generation through a combination of factors: microviscosity, the nature of binding interactions, and the light-scattering effects associated with aggregation.

In summary, this study has employed Thioflavin T (ThT) and three proteins: lysozyme, insulin and α-synuclein to showcase the high sensitivity of lasing to obtain information on microviscosity in the nucleation phase. The entire aggregation process was characterized by steady-state, time-resolved and lasing spectroscopy in α-synuclein protein. This comprehensive approach spans the entire aggregation spectrum, from the dynamics of monomer-to-oligomer transition, oligomer transient states, and finally, to the energetically stable fibrils.

Our findings reveal that weak fluorescence associated with microviscosity changes is subsequently overshadowed by the dye’s confinement within the β-sheets of fibril structures and their grooves. By utilizing lasing spectroscopy, condensating proteins by rising the concentration and taking advantage of ThT being a molecular rotor - a type of dye extensively used in viscosity studies - we have successfully elucidated nonspecific ThT-protein interactions linked with microviscosity in the early stage nucleation phase in three proteins: lysozyme, insulin and α-synuclein where β-sheets are not yet formed. We discovered that alterations in microviscosity facilitate fluorescence enhancement, a phenomenon previously experimentally not reported due to the dominant fluorescence increase caused by dye binding to β-sheet rich amyloid fibrils.

In conclusion, our study demonstrates that lasing spectroscopy can effectively identify early stage intermediate protein structures and transiently populated states during protein aggregation. This capability is attributed to the distinct microenvironments these states provide for the ThT.

## Supporting Information Available

Materials, preparation protocols and methods descriptions; lasing spectra and thresholds for insulin and lysozyme oligomers (PDF)
