# Supplementary material for: Thioflavin T—a Reporter of Microviscosity in Protein Aggregation Process: The Study Case of α-Synuclein
Source: J Phys Chem Lett. 2024 Jun 20;15(25):6685–90. doi: 10.1021/acs.jpclett.4c00699 (PMC11215780; doi:10.1021/acs.jpclett.4c00699)
Supplement: Supplementary file 1 — jz4c00699_si_001.pdf [file jz4c00699_si_001.pdf]

Thioflavin T – Reporter of Microviscosity in Protein Aggregation Process: The Study Case of α-synuclein

K. Rusakov[1], A. El-Turabi[2], L. Reimer[3], P.H. Jensen[3], P. Hanczyc[4]*

[1] Faculty of Construction and Environmental Engineering, Warsaw University of Life Sciences, 02-776, Warsaw, Poland

[2] University of Oxford, Jenner Institute, Nuffield Department of Medicine, OX3 7DQ, Oxford, UK

[3] DANDRITE, Department of Biomedicine, Aarhus University, 8000 Aarhus, Denmark

[4] Institute of Experimental Physics, Faculty of Physics, University of Warsaw, Pasteura 5, 02-093 Warsaw, Poland

Materials and Methods

Materials:

Thioflavin T: was "UltraPure Grade" purchased from AnaSpec (USA). Two stock solutions were prepared: for steady-state and time-resolved fluorescence the stock solution was of 3.14 mM whereas for lasing experiments was between 18.8 mM to 78.4 mM depending on the solvent viscosity (for details see tab 1).

α-synuclein: production and purification was performed on transformed BL21(DE3) competent cells as previously described[1,2] with small modifications. Briefly, transformed bacteria were lysed by sonication, centrifuged for 30 min at 11.000rpm and 4°C using an (F12S-6X500 LEX Sorvall rotor), and the supernatant boiled for 5 minutes to create a heat-soluble bacterial extract. Heat-insoluble proteins were removed by centrifugation at 20.000rpm for 30' and 4°C (F21S-8X50Y Sorvall rotor), and the following protein purification of the heat-soluble supernatant involved dialysis against 20 mM Tris pH 6.5 overnight, followed by ion-exchange chromatography on a Poros HQ50 column (Thermo Fisher Scientific) with a 0–1 M NaCl gradient. Next, a reverse phase-high pressure liquid chromatography purification step was performed on a Jupiter C18 column (Phenomenex, Torrance, CA) in 0.1% trifluoroacetic acid with an 0–90% acetonitrile gradient. Isolated proteins were dialysed in PBS pH 7.4 overnight followed by an additional dialysis step in 20 mM ammonium bicarbonate overnight. Protein concentration was determined by bicinchoninic acid (BCA) protein concentration assay (Pierce). The proteins were subsequently aliquoted, lyophilized, and stored at − 20 °C until use. Human α-syn protein was then dissolved to a final concentration of 12mg/mL (e.g. 1mg in 83.3µl) in 1xPBS and kept on ice. Amicon® Ultra 0.5mL Filters with pore size 100kDa were used for protein purification of pre-aggregates. Centrifugation was conduced in a 40° fixed angle rotor, at temperature 4°C, 14,000 x g. Filtrated solution that passed through the filter was containing only α-synuclein monomers. It was stained with ThT and used for steady-state and time-resolved fluorescence experiments. For lasing experiments α-synuclein monomeric stock was concentrated by ultracentrifugation in Ultra 0.5mL Filters with pore size 3kDa. Using that filter and spinning at 25000 x g, a 10 fold reduction of the starting solution volume was obtained which is corresponding to approximately 120 mg/ml. The concentrate solution was recovered by flipping the filter column and spinning at 1000 x g.

All samples were quaked for 10 min at 900 rpm using thermoshaker (Biosan) in room temperature conditions to initiate α-synuclein aggregation. Each sample for steady-state and time-resolved experiments was transferred to a 15 µL quartz cuvette and heated to 37°C using custom built heating holder. For lasing experiments the 4µL of protein concentrate and 2 µL of ThT stock were mixed and drop casted on the one cavity mirror. Then second cavity mirror was put on top. Sandwiched gain medium (ThT-α-syn) between two mirrors was mounted in a

heating holder. The initial measurement was done in room conditions and the rest measurements were performed at 37°C over time.

Insulin: Insulin monomer protein derived from bovine pancreas was acquired from Sigma-Aldrich. The protein was dissolved to a concentration of 10 mg/ml at pH 2 in 0.003 mM H_2SO_4. For the lasing experiments, the insulin stock solution was concentrated using Ultra 0.5 mL Filters with a 3 kDa pore size. By centrifuging at 25,000 x g, the volume of the starting solution was reduced tenfold, resulting in a concentration of approximately 100 mg/ml. The concentrated solution was then retrieved by inverting the filter column and centrifuging at 1,000 x g.

For the lasing experiments, 4 µL of the insulin solution and 2 µL of ThT stock (78.4 mM) were combined and drop-cast onto one cavity mirror. A second cavity mirror was then placed on top to form a sandwiched gain medium consisting of ThT-insulin between the two mirrors. This assembly was mounted in a heating holder. The sample was heated at 65°C for 30 minutes, after which the lasing signal was collected at 440 nm (Fig S1).

Lysozyme: recombinant human lysozyme protein white was obtained from Sigma-Aldrich. The protein was dissolved to a concentration of 360 mg/ml at pH 2 in 0.003 mM H_2SO_4, resulting in a viscous liquid that indicated an increase in macroviscosity. The solution was then incubated at 65°C for 30 minutes, and lasing spectra were collected as a function of the pump energy.

Methods:

UV-Vis Spectroscopy: Absorption spectra were carried in a cuvette with a 1 cm pathlength in a CARY-5000 spectrophotometer.

Steady-state and time-resolved fluorescence spectroscopy:

For measurements of steady-state and time-resolved fluorescence, femtosecond pulses were generated through frequency doubling in a BBO crystal from the output pulses of an optical parametric amplifier (Orpheus by Light Conversion) pumped by a femtosecond amplifier (Carbide by Light Conversion) at wavelengths of 405 nm or 440 nm. The pulse repetition rate was maintained at 2 MHz. Spectroscopic studies were conducted using a Horiba QuantaMaster 8075-11 spectrofluorometer, equipped with a PPD850 photomultiplier (sensitive in the range of 250-850 nm) and a DeltaTime kit for time-resolved measurements. Emission and excitation spectra were obtained from sample solutions in 1x1 cm quartz cuvettes. Excitation and emission slits were set to 1 nm, and spectra were adjusted for detector sensitivity. The power of the excitation beam was controlled to avoid saturation and ensure a linear detector response. The instrument response function (IRF) was determined by scattering the excitation beam in a TiO_2 suspension in water. Fluorescence decay analysis was carried out using the Horiba FelixGX software, employing a reconvolution method of multiexponential decays with the IRF, and fitting the convoluted functions to the experimental data. The average fluorescence lifetime was calculated as the amplitude-weighted mean of the decay times for each component.

Lasing in cavities: the term refers to Fabry-Perot cavity whereby the liquid with ThT is sandwiched between the two mirrors that act as photonic resonators. The mirror cavity provides strong optical feedback that can be used to detect subtle molecular changes of the ThT microenvironment. Lasing occurs when the excitation energy is gradually increased. It was done by stepwisely moving the grey filter using the Thorlabs motor system and the spectrum of the emitted light was simultaneously monitored in the detector. Plots of the dependence of the pump energy and emission were used to determine the lasing thresholds.

At a given energy the ThT emission spectrum become significantly narrower and a dramatic rise of the light intensity is observed. The pump energy at which that happens is named lasing threshold. Typically, when population inversion occurs in cavity a narrow lasing peak is detected without background fluorescence. The full width at half-maximum (FWHM) of lasing spectrum in cavities is a few nanometers wide.
Lasing spectra were recorded using femtosecond laser working at 0.5 kHz repetition rate and providing energy of a pulse 400 μJ. The output beam was at 800 nm and using the optical amplifier the wavelengths set for experiments were 405 nm and 440 nm.

The mirrors used for cavities had nearly 100% transmission at 400-450 nm. The reflectance in range 470-570 nm was around 95-99%, centered at 520 - 530 nm. The cavity thickness was in range 4 μm – 8 μm for dye in solvents. In case of dye stained proteins the cavity thickness was between 10 – 14 μm.
The lasing signal from the gain medium in cavities was collected parallel to the direction of the excitation beam. A filter was removing excitation >470 nm and only the lasing signal from the sample was reaching the detector. Lasing was collected with a ProEM Excelon camera from Teledyne Princton Instruments.

Supporting figures:

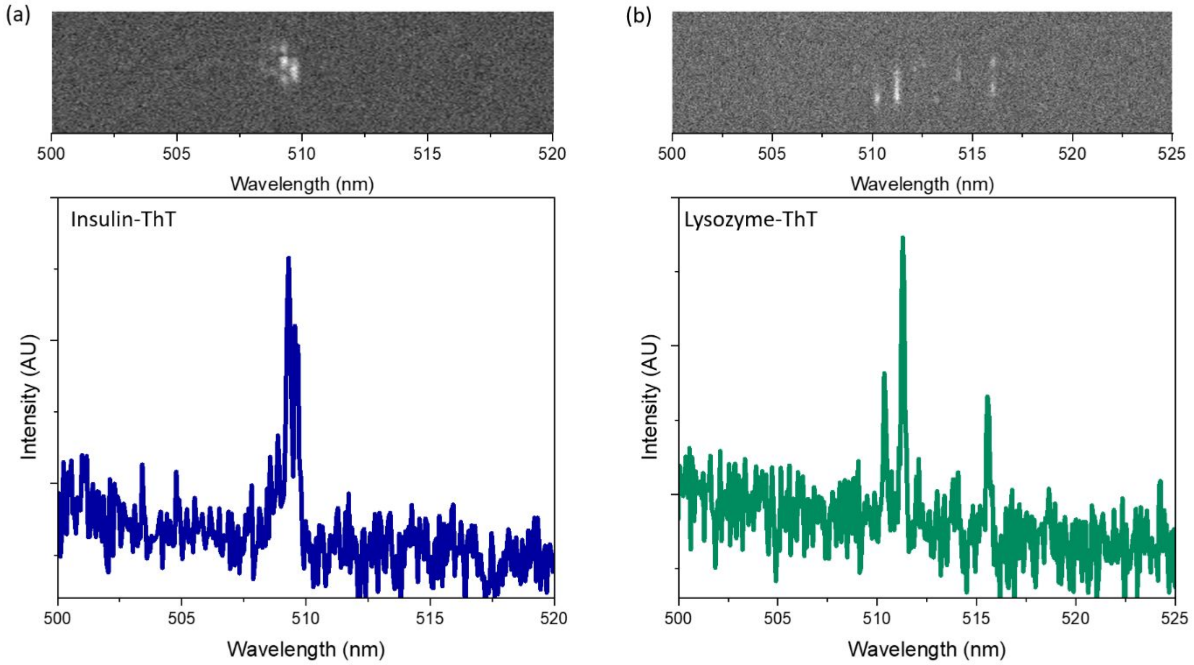

Fig. S1 *Lasing measurement outputs for (a) insulin and (b) lysozyme samples doped with Thioflavin T (ThT), depicted as bright spots in the top panel, demonstrate lasing activity indicative of early-stage protein aggregation. The corresponding lasing spectra are shown in the bottom panel. Samples were heated at 65°C for 30 minutes (excitation wavelength: 405 nm).*

(1) Jensen, P. H.; Hojrup, P.; Hager, H.; Nielsen, M. S.; Jacobsen, L.; Olesen, O. F.; Gliemann, J.; Jakes, R. Binding of Aβ to α-and β-synucleins: Identification of segments in α-synuclein/NAC precursor that bind A β and NAC. *Biochemical Journal* **1997**, *323* (2), 539-546.
(2) Reimer, L.; Haikal, C.; Gram, H.; Theologidis, V.; Kovacs, G.; Ruesink, H.; Baun, A.; Nielsen, J.; Otzen, D. E.; Li, J.-Y. Low dose DMSO treatment induces oligomerization and accelerates aggregation of α-synuclein. *Scientific Reports* **2022**, *12* (1), 3737.
